# Current imaging methods for assessing Graves` orbitopathy activity with particular emphasis on FDG-PET

**DOI:** 10.3389/fendo.2023.1138569

**Published:** 2023-08-03

**Authors:** Anna Ochmann, Mateusz Winder, Joanna Nalewajka-Kołodziejczak, Jerzy Chudek

**Affiliations:** ^1^ Department of Internal Medicine and Oncological Chemotherapy, Medical University of Silesia, Katowice, Poland; ^2^ Department of Radiology and Nuclear Medicine, Medical University of Silesia, Katowice, Poland; ^3^ Nuclear Medicine Department, Medyczne Centrum Diagnostyczne (MCD) Voxel, Katowice, Poland

**Keywords:** positron emission tomography, diagnostic imaging, Graves` orbitopathy, thyroid accompanied orbitopathy, nuclear medicine imaging

## Abstract

The most frequent extrathyroidal Graves’ disease manifestation is Graves’ orbitopathy (GO). The treatment of GO is determined by its severity and activity. There is currently no reliable, impartial method for assessing it clinically or distinguishing fibrosis from active inflammatory disorders. Today, imaging methods including orbital ultrasound (US), computed tomography (CT), and magnetic resonance imaging (MRI) are frequently employed to show pathological abnormalities in the ocular adnexa of GO patients. In addition, a not widely accepted technique – 99mTc-DTPA SPECT – has some potential to evaluate retrobulbar inflammation in GO patients. However, FDG-PET/CT is possibly superior to other imaging modalities in detecting inflammation in GO and it may be useful in assessing disease activity in case of clinical or serological uncertainty. It might also act as an early indicator of GO development and its aggravation before irreversible tissue alterations take place and may be used in the differential diagnosis of inflammatory disorders of the orbit. However, before FDG-PET/CT could be applied in daily clinical practice, the methodology of GO activity assessment with defined cut-off values for radionuclide concentration – standardized units of value (SUV) have to be established and validated. In addition, the limitations of this technique have to be recognized.

## Introduction

One of the most prevalent autoimmune conditions in women is Graves’ disease having the greatest frequency between the ages of twenty and fifty (1, 2). The overall crude incidence of Graves’ disease is estimated at 24.8 per 100,000 population per year (3). The most frequent extrathyroidal Graves’ disease manifestation is Graves’ orbitopathy (GO) which accounts for approximately 60% of all orbital inflammation in the 21-60-year-old population. In individuals with Graves` hyperthyroidism, GO is seen in 25–30% of cases, and it is very infrequently seen in conjunction with hypothyroid autoimmune thyroiditis (4).

Clinical signs of GO include proptosis, inflammation of the conjunctiva and eyelids, and extraocular muscle hypertrophy, which leads to a decrease in ocular mobility and diplopia. Compression of the optic nerves at the orbital apex and loss in visual acuity may occur in the most severe cases (4). Patients with GO will most typically have enlargement of the orbital fatty connective tissues and the extraocular muscle bodies. These alterations are the result of aberrant hyaluronic acid deposition and edema inside these tissues (5). Due to the wide range of symptoms of inflammation and the multiple confounding factors, such as non-inflammatory fibrosis, it is one of the most challenging clinical characteristics of GO to identify (1). Numerous inflammatory illnesses that affect the orbital tissue, such as idiopathic orbital inflammation, infections, systemic disease symptoms in the orbit, primary and secondary orbital neoplasms, and vascular malformations in the orbit, can mimic GO. Most of them are benign but can cause vision loss. Thereby, an accurate evaluation for a proper differential diagnosis is required (4). There is currently no reliable, impartial method for assessing GO clinically or distinguishing fibrosis from active inflammatory disorders (1). A proper diagnosis of the disease’s severity and activity is necessary for effective treatment. Two commonly used grading systems intended to evaluate the activity and severity of GO and direct therapeutic decision-making are the European Group of Graves’ Orbitopathy (EUGOGO) and VISA (vision, inflammation, strabismus, and appearance) classifications (6). The EUGOGO recommends using a seven-point clinical activity scale (CAS) (7). It analyzes the clinical symptoms of retroorbital pain, pain with eye movement, eyelid swelling, eyelid redness, conjunctival redness, conjunctival swelling, and inflammation of the caruncle or plica, each of which is worth one point on the scale. An active form of GO is indicated by the presence of at least three of the seven clinical symptoms listed above. However, neither of the aforementioned methods of GO classification is based on imaging studies, relying solely on clinical features.

## Current imaging methods

Orbital ultrasonography (US), computed tomography (CT), and magnetic resonance imaging (MRI) are imaging methods frequently utilized to show pathological abnormalities in the ocular adnexa of patients with GO (8).

### Orbital ultrasound

The advantages of orbital ultrasonography include its inexpensive cost, short examination time, and lack of radiation. In research by Nagy et al., measurements of the eye muscles and orbital connective tissue taken during US and MRI exams of patients with GO were compared (9). Results showed that US failed to correlate with MRI and did not allow to determine the nature of muscle enlargement, including the activity of the inflammatory process.

Measuring the muscle reflection using A-mode US could allow the identification of patients with moderate-to-severe GO who could benefit from radiotherapy (10). The reflectivity of less than 30% was characterized by high positive predictive value (PPV) of 85% but low negative predictive value (NPV) of 60% with CAS values alone established at 65% and 56% respectively. However, a combination of US (reflectivity ≥ 30%), CAS (< 4), and the duration of the disease (> 18 months) increased the PPV to 79% and the NPV to 89% and thus allowed to identify patients with nonactive disease.

It has been shown that the analysis of blood flow parameters in the ophthalmic artery, superior ophthalmic vein, and central retinal artery using the color Doppler technique correlates with CAS and may be helpful in the differentiation of active from inactive phases of GO (11, 12). However, increased arterial blood flow velocities can be secondary to orbital inflammation of different etiologies.

Although the US alone can be used to diagnose and assess the extent of eye muscle involvement, it cannot make a clear distinction between activity and inactive disease of retrobulbar tissue (9).

### Computed tomography

CT imaging allows a detailed evaluation of the orbit with a great spatial resolution, in a short investigation time with few artifacts, and at a moderate cost (1). CT is particularly helpful when it may be necessary to rule out other orbital pathologies and for demonstrating the compression of the optic nerve at the apex of the orbit (6). The main indications for CT in GO are the assessment of extraocular muscle involvement and the assessment of the degree of exophthalmos (13, 14). The most common intraorbital manifestation of GO is the involvement of a nontendinous portion of extraocular muscles, predominantly inferior and medial recti muscles (RM). The extraocular muscles in GO appear sharply enlarged in a fusiform fashion. CT also allows visualizing the decreased density of the inflamed orbital muscles caused by fat infiltration (15). This has been confirmed by Cohen et al., although their study focused on identifying foci of fatty infiltration within the muscles rather than the mean density of the whole muscle (16). The results obtained by Regensburg et al. on the other hand showed no differences in mean muscle densities in GO patients compared to normal controls (17). They did however find that orbital fat density in GO patients was significantly higher than in healthy individuals. The orbital adipose/connective tissue volume can also be precisely estimated with the aid of a computer program, making orbital CT a recommended study before decompression surgery (7, 18). However, it is impossible to distinguish between active and inactive thyroid-associated orbitopathy (TAO) with CT. Additionally, CT exposes the lens to a large amount of radiation, which over time increases the risk of cataract formation (8, 19).

### Magnetic resonance imaging

When multiple scans are needed to monitor, for example, therapy response, MRI is the preferred modality for orbital imaging. Other benefits include the ability for tissue differentiation, the absence of ionizing radiation, and the ability to detect interstitial edema in individuals with active disease within the RM (8, 19, 20). It has been demonstrated that MRI enables the quantitative evaluation of the extraocular muscles’ thickness and width (21). Additionally, neither the age nor the gender of GO patients has an impact on the ratio of short to long diameter of the expanded muscles (20). The quantification of disease activity, prediction of anti-inflammatory medication response, and the outcome of GO may all be possible using specific MRI sequences (7). While the analysis of the muscle signal intensity in the T2 weighted images without intravenous contrast alone does not allow distinguishing the active from the inactive phase of GO, the contrast-enhanced MRI in T1 weighted images allows such assessment based on the analysis of signal intensity, time to the maximum enhancement and the enhancement and washout ratios (22). Early changes in the course of GO and the monitoring of the response to radiation are both possible with the quantitative measurement of the muscle signal in diffusion-weighted imaging (DWI) and apparent diffusion coefficient (ADC) maps (23). ADC values were also shown to correlate with the CAS of TAO. Nevertheless, it is a more expensive method with a long acquisition time and lower availability, limiting MRI application in daily practice (8). In patients with unilateral or highly asymmetric exophthalmos, suspected optic neuropathy, and euthyroid status with negative thyroid serology, orbital MRI is now advised (7).

## Nuclear medicine in Graves’ orbitopathy

### 99mTc-DTPA SPECT/CT – single-photon emission computed tomography (SPECT) with the use of diethylenetriaminepentaacetic acid tagged with 99mTc (99mTc-DTPA)

Another, although not widely accepted imaging technique is the single-photon emission computed tomography (SPECT), which utilizes 99m-thallium-tagged diethylenetriaminepentaacetic acid (99mTc-DTPA).

In this investigation method, the radiotracer 99mTc-DTPA is administered intravenously (iv) and is then extravasated at the site of inflammation where it combines with the polypeptides of the extracellular inflammatory fluid. The amount of 99mTc-DTPA that builds up at the site of the inflammation, in this case mostly in the oculomotor muscles, is directly correlated with the activity of the infiltrative and edematous process. The procedure makes use of a gamma camera and single-photon emission computed tomography (SPECT) technology to record the gamma radiation generated by the 99mTc combined with DTPA (24).

This method’s relevance was originally mentioned in 2002 by Galuska et al. They demonstrated the possibility of using 99mTc-DTPA SPECT to assess the inflammation of the retrobulbar region in GO patients. When compared to the background, the radiotracer accumulated more in the orbital cavity, which showed the presence of local inflammation. They discovered that the 99mTc-DTPA SPECT OR/B values were substantially greater in the series of orbits with active illness on MRI than in the inactive stage. In this way, they demonstrated that 99mTc-DTPA is a potential tool in the assessment of GO activity. However, they made it clear that a precise assessment of the method’s usefulness was not possible due to the small number of examined patients (only 9) (25).

Authors from the same research institution came to the conclusion that SPECT scintigraphy using 99mTc-DTPA could be an additional diagnostic method useful in determining whether immunosuppressive treatment should be started and in facilitating the assessment of this treatment’s efficacy for treating GO. This study involved 21 patients (26).

Also Szebados in 2013 showed that orbital radiation therapy had an anti-inflammatory impact in GO patients with high orbital DTPA absorption. They also discovered that the irradiation had no positive effect in individuals with initially normal or minimally raised DTPA UV; in certain cases, the treatment could further exacerbate the inflammation. Based on the results of this study and our prior research, DTPA orbital SPECT may be helpful for determining the immunological activity of GO and for choosing patients who will get local radiation and systemic (steroid) immunosuppressive medication or surgical treatment (27).

The authors state that the 99mTc-DTPA scan has many benefits, including a fast procedure duration, low radiation exposure for the patient, low cost (compared to PET-CT), straightforward analysis, and ability to create a spatial visualization of the biological activity of the studied area (24, 25) But there are also a number of drawbacks including the potential risk of repeated investigations (human anti-mouse antibody, or HAMA) reaction, the scan’s relatively low resolution (> 1 cm), as compared to other imaging techniques like CT or MRI (where resolution is 1 mm), which makes it more difficult to interpret the results and does not allow for more accurate identification of the oculomotor muscles that are impacted by inflammation (24, 26).

Szumowski used a hybrid technique called SPECT/CT in his study to assess the type of GO. The SPECT/CT approach offers a significantly more precise image of the accumulated radiotracer location than the classic SPECT method, which increases the accuracy of interpreting the image as either physiological or pathological, depending on its precise location. This is supported by the SPECT/CT method’s high sensitivity and specificity, which were shown in his paper and equal to 93 and 89%, respectively (24). Based on his findings, it can be said that the 99mTc-DTPA SPECT/CT method offers a valuable tool for assessing the active form of GO and is in many ways superior to the standard methods offering just imaging of oculomotor muscles. In conjunction with MRI, it appears that it may be employed in GO as a potential reference diagnostic method (24).

### The principles of FDG-PET imaging

A non-invasive diagnostic technique called positron emission tomography (PET) has been suggested as a method to identify inflammatory and/or malignant processes. It provides the opportunity to perform functional and metabolic assessments in these circumstances, whether or not there has been any structural alteration (28). A radioactive molecular analog of glucose called 18F-fluorodeoxyglucose (FDG) is used in PET imaging as a metabolic marker. The cellular uptake of FDG determines the metabolic activity of the tissues. This approach has the benefit of being “semiquantitative” in terms of radionuclide concentration using standardized units of value (SUV) per gram of the tissue under investigation. The SUVmax value is affected by the patient’s weight, the radionuclide dose, and blood sugar level. This method makes it easier to distinguish between the radionuclide’s pathologic and normal uptake in the studied tissues, providing objective interpretation (28). Some clinicians advocate that lesions with SUVmax greater than 2.5 were considered malignant (29, 30). However, other researchers acknowledge that this strategy has serious flaws that could result in poor patient management (31). SUVmax measurements are affected by many parameters that should be accounted for when using certain FDG PET thresholds to improve the characterization of the disease (31). The influences of competing transport effects, body size, count noise effect, radiotracer dispersion time, partial volume and spillover effects, attenuation correction, reconstruction method, and scanner type all have a significant impact on SUVmax estimations. Inter-institutional SUVmax results are likely to differ as a result of the variation in PET acquisition across various institutions. As a result, diagnostic criteria such as SUVmax of 2.5 that some research groups have reported as effective may not be useful in all other institutions. These values may differ for different scanners and FDG PET acquisition protocols. The radiotracer distribution time is another significant but largely disregarded factor that influences FDG uptake and SUVmax values (i.e. the time between FDG injection and scanning). This is visibly demonstrated in the liver, which shows a 25–30% decrease in SUV between 1 to 2–3 hours (20). As a result, even while focal hepatic activity with SUVmax of 3.5–4.0 on 1-hour PET imaging is typical and considered benign, it may be suggestive of malignancy if PET image acquisition was done 2-3 hours following FDG administration (31). For that reason FDG PET protocol should be normalized and performed at the same established time i.e. after 60 minutes, despite the acceptable time of PET scan is until 90 minutes.

### FDG-PET/CT in GO

The first documented case of GO discovered by FGD-PET/CT was published by Kuo in 2006 (32). In this case, the inflammatory process in GO caused a substantial rise in FDG uptake in both the medial and inferior RM, which was consistent with the patient’s physical findings. The MRI showed only the isolated enlargement of the left inferior RM with spared tendon insertion. This example offered evidence that FDG-PET/CT may be more sensitive than MRI for detecting inflammation in GO. It also supports the results of other studies that compared MRI and PET’s capabilities in imaging inflammation (32). The results of FDG-PET and MRI in the diagnosis of aortitis were comparable, according to Meller’s report from 2003, however, FDG-PET imaging revealed more vascular regions engaged in the inflammatory process (18).

A study by Nakamura et al. compared the various physiological FDG accumulations in the head and neck area with pathological FDG accumulation caused by tumors. It might be challenging to predict malignancy on PET imaging because physiological FDG accumulation can often be confused with pathological accumulation in normal tissues of the head and neck. According to the study, the sublingual gland, extraocular muscle, and tonsil showed relatively high physiological cut-off values for SUVmax with poor diagnostic accuracy. For extraocular muscle, SUVmax of 10.0 was considered the optimal cut-off value for differentiating tumor from physiological accumulation. A SUVmax of ≥ 1.5 or the presence of a significant metabolic asymmetry is highly suspicious of a tumor (33).

The prospective study by Garcia-Rojas et al. was the first to describe the physiological FDG uptake levels in extraocular muscles and to compare them with patients with Graves’ ophthalmopathy. Patients with and without GO showed statistically different FDG uptake of the extraocular muscles (*p* 0.05). FDG-PET/CT might be helpful in determining the inflammatory status when a clinical or serological dispute exists because there is no objective technique to distinguish the active inflammatory alterations from fibrosis. Results indicated that PET/CT may be a valuable imaging technique for the accurate identification of inflammatory as well as morphological orbital abnormalities in GO patients (28).

The same author proved a lack of correlation between 18-FDG extraocular muscle uptake and either the clinical inflammation score or muscle diameter in research published the following year (28). 18-FDG uptake is a sensitive marker of inflammation, however in GO inflammation may be clinically present in PET/CT-negative cases, but also patients with negative clinical findings may show inflammation in PET/CT (1, 18). Although the strength and size of the sample in this study limited the conclusion that there was no link, a statistically significant difference in the degree of inflammation determined clinically using the VISA classification and FDG-PET was not identified. The various onset periods of the patients’ hyperthyroidism and ophthalmopathy, as well as their varied hormonal conditions, were additional limitations. 18-FDG PET may detect cases in which clinical assessment is confusing and VISA is not precise. Morphological changes are completely independent of the degree of inflammatory activity. The morphological study is useful for evaluating orbital anatomy, but according to this study, it may not determine the inflammatory status of the orbital structures. Still, as previously published by his group, patients with GO did have increased FDG uptake compared with patients without GO (1, 28).

According to a 2017 study by Uslu-Beşli, orbital FDG-PET/CT may be able to detect orbitopathy in Graves’ disease earlier than MRI or CT. The extraocular muscle uptake in patients with Graves’ disease was increased regardless of muscle thickness, suggesting that FDG PET/CT may be able to detect inflammation in extraocular muscles before morphological alterations take place (34). This finding potentially facilitates the selection of individuals with active inflammation in the extraocular muscles, which is crucial for treatment planning. Extraocular muscle uptake was not seen after corticosteroid prophylaxis administration to GO patients following radioiodine treatment. Radioiodine therapy was shown to be a risk factor for the development or worsening of orbitopathy in patients without prophylaxis. Smoking was another independent risk factor for the onset of GO. When SUVmax values for each RM were evaluated before and after radioiodine therapy, smokers did not exhibit any changes in the SUVmax values. However, there was a statistically significant SUVmax increase in all RMs in the nonsmoker group. The study’s weaknesses included the small number of patients and the absence of MRI correlation, which is considered the modality of choice for GO detection (34).

The retrospective study by Elourimi et al. was the first to examine at the potential use of FDG-PET/CT for the etiological workup of orbital inflammatory disorders (IOD). OID may be the ophthalmological manifestation of systemic disorders (SD), such as Graves’ disease, adult-onset asthma and periorbital xanthogranuloma (AAPOX), sarcoidosis, granulomatosis with polyangiitis (GPA), lymphoma, crystal-storing histiocytosis (CSH), or IgG4-related disease (IgG4-RD). This research offers proof that nuclear imaging might be useful for diagnosing underlying (but undiscovered) systemic disease and for accurate disease staging at diagnosis. The researchers advise undertaking an FDG-PET/CT as a second-line test if the results of the first line of studies are negative (35). **Supplementary Figures 1** and **2** present MRI and PET/CT images of a patient with GO treated at our Clinic.

### FDG-PET/MRI

The combination of PET and MRI utilizes the advantages of both highly sensitive modalities in GO diagnostics.

Weber et al. confirmed that FDG-PET/MRI can evaluate the disease activity in patients with GO and might prove beneficial for the distinction of mild vs. moderate-to-severe vs. sight-threatening GO in combination with the clinical assessment. It could also help to identify the patients who require more aggressive treatment. The limitation of PET/MRI imaging was that it lasts longer and requires a precise setting to minimize eye motion artifacts. PET/MRI is currently not recommended for clinical use but it can serve as an academic tool to explain the pathophysiology of GO. Technical progress towards shorter protocols and new tracers that are less sensitive to confounding might enable the diagnosis of GO in PET/MRI in the future (36).

## A new direction for PET in Graves’ orbitopathy diagnostics

In 2019 Laban proposed the innovative use of Zirconium-89 labeled PET/CT imaging of GO as a diagnostic method to select potential patients that might benefit from rituximab treatment used i.e. rheumatoid arthritis. The study showed that 3 out of 4 patients with intense 89Zr-rituximab uptakes in orbital inflammatory disease responded well to rituximab treatment (37). Bart de Keizer et al. also presented a case report where they described a patient with GO resistant to intravenous glucocorticoids (GCs). PET-CT performed 3 days after 89Zr-rituximab showed high uptake in thickened medial RM of the left eye with SUVmax of 5.9 and the superior RM of the right eye with SUVmax of 5.2. Because of the high 89Zr-rituximab uptake, the patient received rituximab treatment (38).

^89^Zr-rituximab-PET has the potential to detect B cell-mediated disease within the orbit and ocular adnexa. It may also help to distinguish the inflammatory and lymphoproliferative disorders from other orbital diseases and to select the patients with orbital inflammatory disease (including GO) that might benefit from rituximab treatment (38).

A case report by Pichler et al. demonstrated the possible utility of gallium (Ga) labeled PET/CT in GO diagnosis (39). The ^68^Ga-labeled [1, 4, 7, 10-tetraazacyclododecane-1, 4, 7, 10-tetraacetic acid]-1-NaI3-octreotide (DOTA-NOC) PET/CT, routinely used to detect the somatostatin receptors in neuroendocrine tumors (NET), was performed in the patient presenting with chemosis, eye paresis and laboratory markers of GO. The assumption of this method is based on the expression of somatostatin receptors found on activated T lymphocytes (40). The examination showed increased accumulation of the radiotracer in the thyroid and single inferior RM, representing active orbitopathy.

The summary of the available FDG/PET research is presented in **Supplementary Table 1**.

## Summary and conclusions

Imaging studies frequently leave doubts about the persistence of GO activity after treatment.

They allow the exclusion of significant muscle involvement by the inflammatory process in symptomatic patients. However, in doubtful cases the assessment of activity with SPECT or FGF-PET can be a valuable diagnostic tool in the decision-making process, limiting the use of often very costly for health systems and burdensome for the patient therapies.

Of note, the presence of muscle damage, imaging studies are not sensitive enough to assess GO activity. Assessment of local inflammation can be based on FDG-PET/CT and also the new methods correlated with FDG-PET/CT, such as Zirconium-89 labeled PET-CT or FDG-PET/MRI likewise, the less common available technique 99mTc-DTPA SPECT. They can be an be a viable tool in GO activity evaluation, treatment method determination, and treatment efficacy assessment, although they all need more accurate confirmation on a bigger group of patients.

The problem in the FDG-PET/CT evaluation remains the methodology of GO activity assessment with defined cut-off values for radionuclide concentration and the appropriate period of time after the end of therapy, when a follow-up examination should be performed. Standardized units of value (SUV) have to be established and validated, before this method would be considered in daily clinical practice.

## Author contributions

AO drafted the article, collected the data, analyzed and made interpretations of the data. MW revised the article, took part in the acquisition of data, analyzed and made interpretation of data and prepared figures. JN-K revised the article and made final approval of the version to be published. The work was overseen by JC, who carried out the data analysis and revised the manuscript.

## References

[1] García-Rojas L Adame-Ocampo G Mendoza-Vázquez G Alexánderson E Tovilla-Canales JL. Orbital positron emission tomography/computed tomography (PET/CT) imaging findings in graves ophthalmopathy. BMC Res Notes (2013) 6:353. doi: 10.1186/1756-0500-6-353 24007404 PMC3766662

[2] Pokhrel B Bhusal K. Graves’ Disease. 2022. In: StatPearls. Treasure Island (FL: StatPearls Publishing (2022). 28846288

[3] Hussain YS Hookham JC Allahabadia A Balasubramanian SP. Epidemiology, management and outcomes of Graves’ disease—real life data. Endocrine (2017) 56:568–78. doi: 10.1007/s12020-017-1306-5 PMC5435772 28478488

[4] Marinò M Ionni I Lanzolla G Sframeli A Latrofa F Rocchi R. Orbital diseases mimicking graves’ orbitopathy: a long-standing challenge in differential diagnosis. J Endocrinol Invest. (2020) 43:401–11. doi: 10.1007/s40618-019-01141-3 31691261

[5] Garrity JA Bahn RS. Pathogenesis of graves ophthalmopathy: implications for prediction, prevention, and treatment. Am J Ophthalmol (2006) 142:147–53. doi: 10.1016/j.ajo.2006.02.047 PMC3960010 16815265

[6] Barrio-Barrio J Sabater AL Bonet-Farriol E Velázquez-Villoria Á Galofré JC. Graves’ ophthalmopathy: VISA versus EUGOGO classification, assessment, and management. J Ophthalmol (2015) 2015:249125. doi: 10.1155/2015/249125 26351570 PMC4553342

[7] Bartalena L Kahaly GJ Baldeschi L Dayan CM Eckstein A Marcocci C. The 2021 European Group on Graves' orbitopathy (EUGOGO) clinical practice guidelines for the medical management of Graves' orbitopathy. Eur J Endocrinol (2021) 185:G43–67. doi: 10.1530/EJE-21-0479 34297684

[8] Kahaly GJ. Imaging in thyroid-associated orbitopathy. Eur J Endocrinol (2001) 145:107–18. doi: 10.1530/eje.0.1450107 11454505

[9] Nagy EV Toth J Kaldi I Damjanovich J Mezosi E Lenkey A. Graves’ ophthalmopathy: eye muscle involvement in patients with diplopia. Eur J Endocrinol (2000) 142:591–7. doi: 10.1530/eje.0.1420591 10832104

[10] Gerding MN Prummel MF Wiersinga WM. Assessment of disease activity in Graves' ophthalmopathy by orbital ultrasonography and clinical parameters. Clin Endocrinol (Oxf). (2000) 52:641–6. doi: 10.1046/j.1365-2265.2000.00973.x 10792345

[11] Yanik B Conkbayir I Acaroglu G Hekimoglu B. Graves' ophthalmopathy: comparison of the Doppler sonography parameters with the clinical activity score. J Clin Ultrasound (2005) 33:375–80. doi: 10.1002/jcu.20154 16240428

[12] Alp MN Ozgen A Can I Cakar P Gunalp I. Colour Doppler imaging of the orbital vasculature in Graves' disease with computed tomographic correlation. Br J Ophthalmol (2000) 84:1027–30. doi: 10.1136/bjo.84.9.1027 PMC1723625 10966959

[13] Gonçalves AC Gebrim EM Monteiro ML. Imaging studies for diagnosing Graves' orbitopathy and dysthyroid optic neuropathy. Clinics (Sao Paulo) (2012) 67:1327–34. doi: 10.6061/clinics/2012(11)18 PMC3488994 23184212

[14] Fang ZJ Zhang JY He WM. CT features of exophthalmos in Chinese subjects with thyroid-associated ophthalmopathy. Int J Ophthalmol (2013) 18:146–9. doi: 10.3980/j.issn.2222-3959.2013.02.07 PMC3633750 23638413

[15] Ozgen A Alp MN Ariyürek M Tütüncü NB Can I Günalp I. Quantitative CT of the orbit in Graves' disease. Br J Radiol (1999) 72:757–62. doi: 10.1259/bjr.72.860.10624341 10624341

[16] Cohen LM Liou VD Cunnane ME Yoon MK. Radiographic analysis of fatty infiltration of the extraocular muscles in thyroid eye disease. Orbit (2022) 41:53–8. doi: 10.1080/01676830.2020.1817100 32878536

[17] Regensburg NI Wiersinga WM Berendschot TT Saeed P Mourits MP. Densities of orbital fat and extraocular muscles in graves orbitopathy patients and controls. Ophthalmic Plast Reconstr Surg (2011) 27:236–40. doi: 10.1097/IOP.0b013e31820365d5 21242855

[18] Meller J Strutz F Siefker U Scheel A Sahlmann CO Lehmann K. Early diagnosis and follow-up of aortitis with [18F]FDG PET and MRI. Eur J Nucl Med Mol Imaging (2003) 30:730–6. doi: 10.1007/s00259-003-1144-y 12677302

[19] Müller-Forell W Kahaly GJ. Neuroimaging of graves’ orbitopathy. Best Pract Res Clin Endocrinol Metab (2012) 26:259–71. doi: 10.1016/j.beem.2011.11.009 22632363

[20] Aydin K Güven K Sencer S Cıkım A Gül N Minareci O. A new MRI method for the quantitative evaluation of extraocular muscle size in thyroid ophthalmopathy. Neuroradiology (2003) 45:184–7. doi: 10.1007/s00234-002-0930-8 12684723

[21] Chen Y Jin ZY Zhang ZH Xu DD MW Jiang B Fang HY. Quantitative evaluation of extraocular muscle with high-field magnetic resonance in patients with Graves’ ophthalmopathy with upper-lid retraction. Zhongguo Yi Xue Ke Xue Yuan Xue Bao. (2012) 34:461–7. doi: 10.3881/j.issn.1000-503X.2012.05.005 23134821

[22] Jiang H Wang Z Xian J Li J Chen Q Ai L. Evaluation of rectus extraocular muscles using dynamic contrast enhanced MR imaging in patients with Graves’ ophthalmopathy for assessment of disease activity. Acta Radiol (2012) 53:87–94. doi: 10.1258/ar.2011.110431 22184678

[23] Yu W Zheng L Shuo Z Xingtong L Mengda J Lin Z. Evaluation of extraocular muscles in patients with thyroid associated ophthalmopathy using apparent diffusion coefficient measured by magnetic resonance imaging before and after radiation therapy. Acta Radiol (2022) 63:1180–6. doi: 10.1177/02841851211034042 34338029

[24] Szumowski P Abdelrazek S Żukowski Ł Mojsak M Sykała M Siewko K. Efficacy of 99mTc-DTPA SPECT/CT in diagnosing Orbitopathy in graves' disease. BMC Endocr Disord (2019) 19:10. doi: 10.1186/s12902-019-0340-0 30658624 PMC6339418

[25] Galuska L Leovey A Szucs-Farkas Z Garai I Szabo J Varga J. SPECT using 99mTc-DTPA for the assessment of disease activity in Graves' ophthalmopathy: a comparison with the results from MRI. Nucl Med Commun (2002) 23:1211–6. doi: 10.1097/00006231-200212000-00010 12464787

[26] Galuska L Leovey A Szucs-Farkas Z Szabados L Garai I Berta A. Imaging of disease activity in Graves' orbitopathy with different methods: comparison of (99m)Tc-DTPA and (99m)Tc-depreotide single photon emission tomography, magnetic resonance imaging and clinical activity scores. Nucl Med Commun (2005) 26:407–14. doi: 10.1097/00006231-200505000-00003 15838422

[27] Szabados L Nagy EV Ujhelyi B Urbancsek H Varga J Nagy E. The impact of 99mTc-DTPA orbital SPECT in patient selection for external radiation therapy in Graves' ophthalmopathy. Nucl Med Commun (2013) 34:108–12. doi: 10.1097/MNM.0b013e32835c19f0 23196678

[28] García-Rojas L Adame-Ocampo G Alexánderson E Tovilla-Canales JL. 18-Fluorodeoxyglucose uptake by positron emission tomography in extraocular muscles of patients with and without graves’ ophthalmology. J Ophthalmol (2013) 2013:529187. doi: 10.1155/2013/529187 23476741 PMC3586501

[29] Hain SF Curran KM Beggs AD Fogelman I O’Doherty MJ Maisey MN. FDG-PET as a “metabolic biopsy” tool in thoracic lesions with indeterminate biopsy. Eur J Nucl Med (2001) 28:1336–40. doi: 10.1007/s002590100563 11585292

[30] Beggs A Hain S Curran K O’Doherty M. FDG-PET as a “metabolic biopsy” tool in non-lung lesions with indeterminate biopsy. Eur J Nucl Med (2002) 29:542–6. doi: 10.1007/s00259-001-0736-7 11914894

[31] Kwee TC Cheng G Lam MGEH Basu S Alavi A. SUVmax of 2.5 should not be embraced as a magic threshold for separating benign from malignant lesions. Eur J Nucl Med Mol Imaging. (2013) 40:1475–7. doi: 10.1007/s00259-013-2484-x 23801170

[32] Kuo PH Monchamp T Deol P. Images in thyroidology* Imaging of inflammation in graves’ Ophthalmopathy by positron emission tomography/computed tomography. Thyroid (2006) 16:419–20. doi: 10.1089/thy.2006.16.419 16646690

[33] Nakamura S Okochi K Murata Y Shibuya H Kurabayashi T. [18F]Fluorodeoxyglucose-PET/CT differentiation between physiological and pathological accumulations in head and neck. Nucl Med Commun (2009) 30:498–503. doi: 10.1097/MNM.0b013e3283299a52 19434008

[34] Uslu-Beşli L Kabasakal L Sağer S Cicik E Asa S Sönmezoğlu K. Orbital flourine-18-fluorodeoxyglucose positron emission tomography in patients with Graves’ disease for evaluation of active inflammation. Nucl Med Commun (2017) 38:964–70. doi: 10.1097/MNM.0000000000000737 28863123

[35] Elourimi G Soussan M Groh M Martin A Héran F Galatoire O. F-18 fluorodeoxyglucose PET/CT as a diagnostic tool in orbital inflammatory disorders. OcularImmunol Inflammation (2022) 30:1803–9. doi: 10.1080/09273948.2021.1957943 34319821

[36] Weber M Deuschl C Bechrakis N Umutlu L Antoch G Eckstein A. 18 F-FDG-PET/MRI in patients with Graves’ orbitopathy. Graefes Arch Clin Exp Ophthalmol (2021) 259:3107–17. doi: 10.1007/s00417-021-05339-1 PMC8478760 34406498

[37] Laban KG Kalmann R Leguit RJ de Keizer B. Zirconium-89-labelled rituximab PET-CT in orbital inflammatory disease. EJNMMI Res (2019) 9:68. doi: 10.1186/s13550-019-0530-9 31363937 PMC6667535

[38] de Keizer B Laban KG Kalmann R. Zirconium-89 labelled rituximab PET-CT imaging of Graves’ orbitopathy. Eur J Nucl Med Mol Imaging. (2020) 47:738–9. doi: 10.1007/s00259-019-04599-8 PMC7005082 31741019

[39] Pichler R Sonnberger M Dorninger C Assar H Stojakovic T. Ga-68-DOTA-NOC PET/CT reveals active graves’ Orbitopathy in a single extraorbital muscle. Clin Nucl Med (2011) 36:910–1. doi: 10.1097/RLU.0b013e31821a2618 21892044

[40] Pasquali D Notaro A Bonavolonta’ G Vassallo P Bellastella A Sinisi AA. Somatostatin receptor genes are expressed in lymphocytes from retroorbital tissues in Graves’ disease. J Clin Endocrinol Metab (2002) 87:5125–9. doi: 10.1210/jc.2002-020790 12414882

