# Supplementary material for: Current imaging methods for assessing Graves` orbitopathy activity with particular emphasis on FDG-PET
Source: Front Endocrinol (Lausanne). 2023 Aug 3;14:1138569. doi: 10.3389/fendo.2023.1138569 (PMC10435873; doi:10.3389/fendo.2023.1138569)
Supplement: Supplementary file 1 [file Table_1.pdf]

Supplementary Material

Current imaging methods for assessing Graves` orbitopathy activity with particular emphasis on FDG-PET

Anna Ochmann [1*] ***, Mateusz Winder*** [2] ***, Joanna Nalewajka-Kołodziejczak*** [3] ***, Jerzy Chudek*** [1]

[1] Department of Internal Medicine and Oncological Chemotherapy, Medical University of Silesia, 40-028 Katowice, Poland

[2] Department of Radiology and Nuclear Medicine, Medical University of Silesia, 40-055 Katowice, Poland

[3] Nuclear Medicine Department MCD Voxel, 40-514 Katowice, Poland

*** Adress for correspondence:**
Anna Ochmann
e-mail: anna.ochmann@interia.pl

Keywords: Positron emission tomography, PET/CT, diagnostic imaging, Graves` orbitopathy, thyroid accompanied orbitopathy

Supplementary Material

Table 1. The summary of the FDG/PET research.

**FDG-PET researcher (year)**	**Number of patients**	**Severity of orbitopathy**	**Advantages**	**Disadvantages**
Kuo (2006)	1 W	1 severe	The sensitivity of FDG-PET/CT for the detection of inflammation in GO may be superior to MRI.	Single case.
Nakamura (2009)	112 (78 M, 34 W)	-	Physiological tonsil, extraocular muscle, and sublingual gland showed relatively high FDG accumulation (sometimes similar to tumor accumulation) – the optimal cut-off values of SUVmax for differentiating tumor from physiological accumulation were considered to be 10.0 for extraocular muscle, The right-to-left ratio of SUVmax was considered useful in differentiating tumor from physiological accumulation (the presence of tumor might be highly suspected in cases with a ratio of 1.5 or more).	Lack of GO severity assessment. Delayed phase PET images only.
Garcia-Rojas (2013)	16 with GO (10 W, 6 M), 35 without GO (18 W, 17 M).	-	The sensitivity of FDG-PET/CT in detecting inflammation in GO is possibly superior to CT alone and even MRI. FDG-PET/CT may be useful in	Small sample size.

			assessing inflammatory status when clinical and/or serological doubt exists.<br>PET/CT may be useful as an accurate imaging method to characterize not only morphological but also inflammatory orbital findings in patients with GO.	
Garcia-Rojas (2013)	16 with GO (10 W, 6 M)	11 mild<br>18 moderate<br>3 severe	No statistically significant difference (*p* = 0.09) in the degree of inflammation assessed clinically by means of the VISA classification and FDG-PET,<br>18-FDG may detect cases in which clinical assessment is confusing and VISA is not precise,<br>Morphological changes are completely independent of the degree of inflammatory activity	Small sample size.<br>Varied hormonal status and time of ophthalmopathy onset.
Uslu-Beşli (2017)	31 with GO (20 W, 11 M), 17 without GO (8 W, 9 M)	-	FDG PET/CT showed increased extraocular muscle uptake in patients with GO.<br>Extraocular muscle FDG uptake was found to be irrelevant from muscle thickness in all RMs in GO group,<br>When SUVmax values for each RM before and after the radioiodine therapy were	Small sample size.<br>Lack of MRI correlation (current modality of choice for GO).

			compared, smokers did not show any increase in the SUVmax values in any of the RMs, although there was a statistically significant increase in all RMs in the nonsmoker group. Smoking was shown to be an independent risk factor for the development of GO.<br>Extraocular muscle uptake does not increase in GO patients after radioiodine when corticosteroid prophylaxis is given.	
Elourmi (2021)	22 (14 W, 8M)	8 patients with idiopathic orbital inflammatory syndrome (OIOS)	A significant fraction of OID patients whose systemic manifestation would have gone undiagnosed with standard etiological workup could be accurately reclassified using FDG-PET/CT, making it a crucial diagnostic tool in the process. A life-threatening condition that was initially misdiagnosed was diagnosed with remarkable success using FDG-PET/CT.	Small sample size. Because the study was carried out in a tertiary referral academic center, there may have been a selection bias.
Weber (2021)	14 with GO (11 W, 3 M)	3 mild<br>7 moderate-to-severe<br>4 sight-threatening	^{18}F-FDG-PET/MRI might be a useful method to precisely assess the inflammation of different tissues in GO.	Small sample size, precluding statistically significant differences in PET

			It might be beneficial for the distinction of mild vs. moderate-to-severe vs. sight-threatening GO and the identification of patients who require more aggressive treatments,<br>It might be beneficial to accurately assess and monitor the inflammation which might be challenging with current methods.	parameters when comparing patients with mild vs. moderate GO,<br>PET/MRI is a lengthy examination and needs a very precise setting to minimize confounding of the examination by eye movements.
Laban (2019)	12 (2 with GO, 8 with idiopathic orbital inflammation,<br>1 with IgG4+ ROD,<br>1 patient with an optic nerve meningioma)	-	^{89}Zr-rituximab PET/CT has the potential to be a powerful tool for the detection of B cell-mediated disease within the orbit and ocular adnexa,<br>Patients with a strong 89Zr-rituximab PET/CT uptake responded well to rituximab treatment,<br>89Zr-rituximab PET/CT can be of aid in distinguishing inflammatory and lymphoproliferative disorders from other orbital diseases.	Small sample size.<br>Not all patients were treated with rituximab, including the patients with a negative scan. Comparison of patients with a positive and negative scan for treatment effectivity was not possible
Bart de Keizer (2020)	1 GO refractory to intravenous GCs	-	^{89}Zr-rituximab PET scanning can help in selecting patients with orbital inflammatory disease	Single case.

			(including GO) that might benefit from rituximab treatment.	
Pichler (2011)	1 W	-	The expression of somatostatin receptors on activated T-lymphocytes can be measured by somatostatin-receptor scan. Thus, a positive orbital octreoscan or alternatives indicate clinically active eye disease in which immunosuppressive treatment might be of therapeutic benefit.	Single case.

GO - Graves` orbitopathy; M – men; W – women
