# Supplementary material for: Current imaging methods for assessing Graves` orbitopathy activity with particular emphasis on FDG-PET
Source: Front Endocrinol (Lausanne). 2023 Aug 3;14:1138569. doi: 10.3389/fendo.2023.1138569 (PMC10435873; doi:10.3389/fendo.2023.1138569)
Supplement: Supplementary file 2 [file Image_1.pdf]

Supplementary Material

Current imaging methods for assessing Graves` orbitopathy activity with particular emphasis on FDG-PET

Anna Ochmann[1*], Mateusz Winder[2], Joanna Nalewajka-Kołodziejczak[3], Jerzy Chudek[1]

[1] Department of Internal Medicine and Oncological Chemotherapy, Medical University of Silesia, 40-028 Katowice, Poland

[2] Department of Radiology and Nuclear Medicine, Medical University of Silesia, 40-055 Katowice, Poland

[3] Nuclear Medicine Department MCD Voxel, 40-514 Katowice, Poland

*** Adress for correspondence:**
Anna Ochmann
e-mail: anna.ochmann@interia.pl

Keywords: Positron emission tomography, PET/CT, diagnostic imaging, Graves` orbitopathy, thyroid accompanied orbitopathy

Supplementary Material

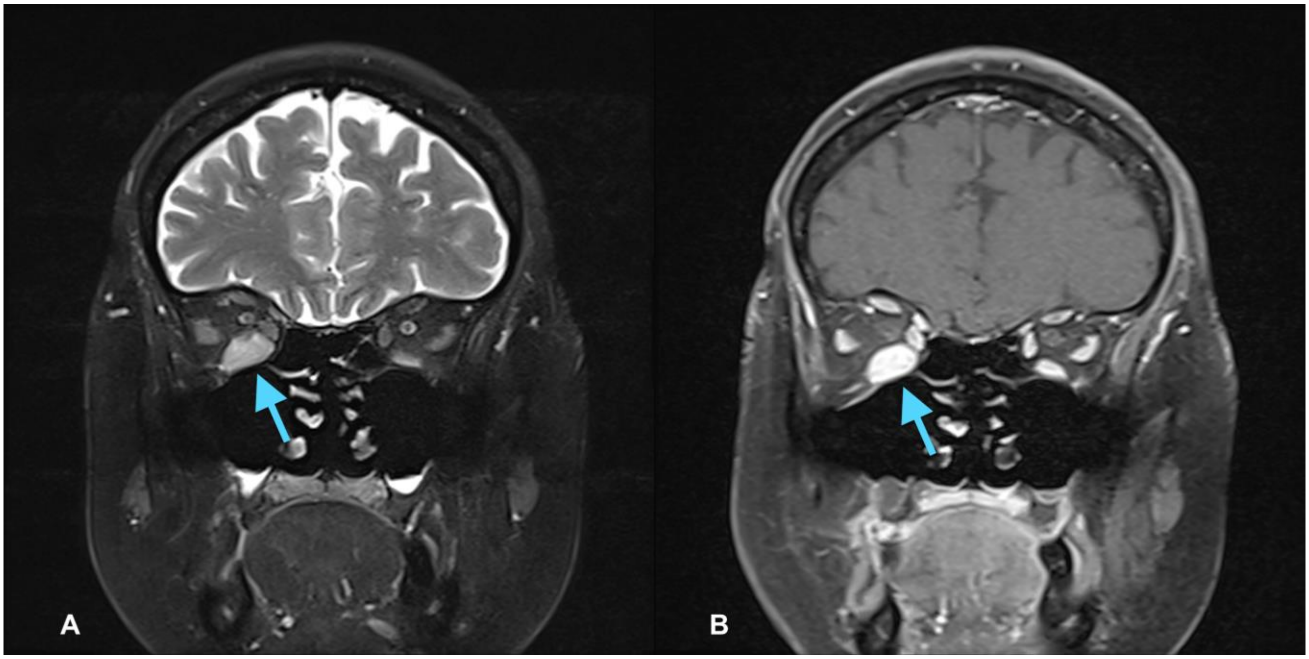

Figure 1. MRI in coronal plane of a 60 year old patient with GO shows enlarged right inferior RM with increased signal in T2W (A) and contrast enhancement in fat saturated (FS) contrast enhanced T1W sequence (B).

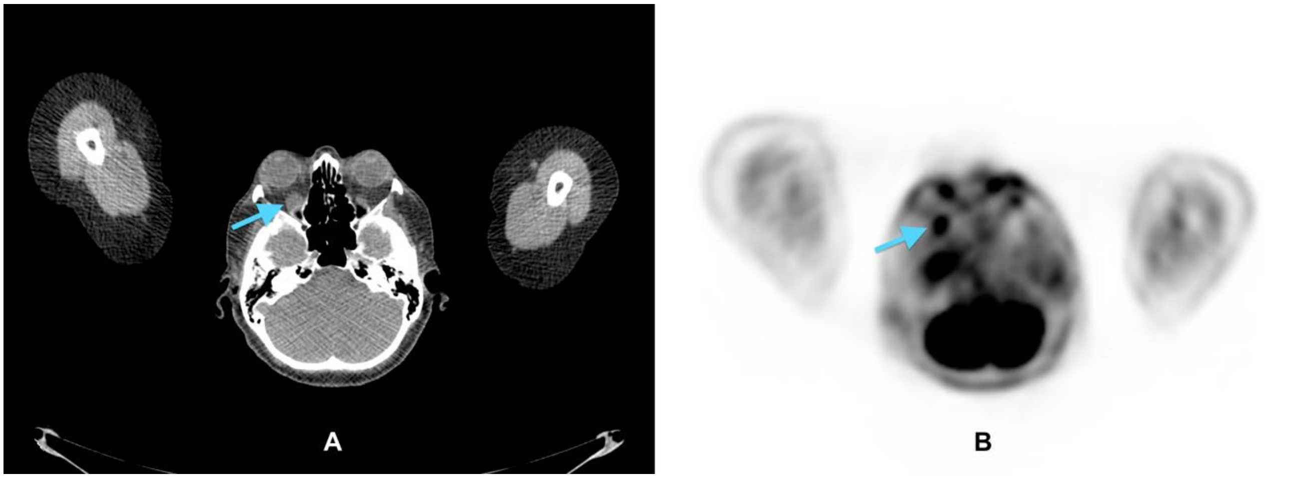

Figure 2. PET/CT of the same patient showing enlarged body of the right inferior RM in transverse low-dose CT (A) and increased uptake of the radio-tracer within the muscle in PET scan (B).
